# Bradycardia, Renal Failure, Atrioventricular Nodal Blockade, Shock, and Hyperkalemia Syndrome due to Amlodipine: A Case Report of an Underdiagnosed Medical Condition

**DOI:** 10.7759/cureus.21144

**Published:** 2022-01-12

**Authors:** Rita Gouveia, Hugo Veiga, Ana A Costa, Joana Pereira, Patrícia Lourenço

**Affiliations:** 1 Department of Internal Medicine, Centro Hospitalar Universitário de São João, Porto, PRT

**Keywords:** drug-induced bradycardia, hyperkalemia, av block, shock, renal failure, amlodipine

## Abstract

We report the case of an 89-year-old female patient who presented to the emergency department with BRASH syndrome, an acronym that stands for bradycardia, renal failure, atrioventricular nodal blockade, shock, and hyperkalemia, which is an underdiagnosed and recently described clinical entity. Contrary to either hyperkalemia or atrioventricular nodal blockade alone, this syndrome represents the synergistic combination of both together, creating a vicious cycle. Conservative treatment of each component, avoiding invasive measures like dialysis or pacing, usually leads to complete resolution. Recognizing the existence of this syndrome is important for an integrative approach and to avoid its recurrence. The association between BRASH syndrome and amlodipine, a dihydropyridine calcium channel blocker, is not commonly described in literature.

## Introduction

BRASH syndrome, an acronym that stands for bradycardia, renal failure, atrioventricular nodal blockade, shock, and hyperkalemia, has been recently described and recognized as a proper entity [1-5]. Contrarily to either hyperkalemia or atrioventricular nodal blockade alone, this syndrome represents the synergistic combination of both together, creating a vicious cycle. Considering the management of these two components is crucial to achieving the best patient outcome [2,4,5].

## Case presentation

Our patient is an 89-year-old female with a medical history of essential hypertension treated with amlodipine 10 mg oral daily and furosemide 40 mg oral daily, type 2 diabetes mellitus treated with neutral protamine Hagedorn (NPH) insulin, paroxysmal atrial fibrillation (AF) anticoagulated with edoxaban 60 mg once daily, and hypothyroidism due to autoimmune thyroiditis treated with levothyroxine 112 mcg once daily. She had been previously hospitalized (in January 2019) due to prostration and sinus bradycardia, the electrocardiogram (EKG) presenting a first-degree AV blockade and right bundle branch block (RBBB). On that previous hospitalization, hyponatremia of 125 mEq/L and hyperkalemia of 5.7 mEq/L were documented; free T4 was normal (1.18 ng/mL), and thyroid-stimulating hormone (TSH) was slightly elevated (5.43 µIU/mL); glucose was 216 mg/dL, and plasma creatinine was 1.34 mg/dL (basal value of 0.8 mg/dL); during this time, the patient has been treated with bisoprolol 5 mg once daily. After beta-blocker (BB) washout and correction of ion disorders, the EKG normalized, and she had a full recovery of her mental status. At discharge, she was medicated with amlodipine 10 mg od, and BB was suspended.

Two years later, in January 2021, the patient was admitted again due to prostration. She was hypoglycemic (52 mg/dL), hypothermic (33.2ºC), and bradycardic (50 beats per minute), and her blood pressure was 132/54 mmHg. Arterial blood gas with FiO_2_ 21% documented a respiratory acidemia: pH 7.327, pCO_2_ 53.5 mmHg, pO_2_ 73.6 mmHg, and HCO_3_-act 27 mmol/L. EKG revealed sinus bradycardia. Laboratory test results showed normal sodium (137 mEq/L), hyperkalemia (5.6 mEq/L), glucose of 157 mg/dL, plasma creatinine of 1.2 mg/dL, and a C-reactive protein (CRP) elevation (18 mg/L). Passive rewarming was done, and warm intravenous normal saline was given. She presented nitrituria and leukocyturia on the urinalysis, and a final diagnosis of urinary tract infection was established. Although this was most likely a coincidental finding, she was discharged on antibiotherapy.

Two weeks later, she presented again at the hospital due to a postprandial syncope. She has had aqueous diarrhea before, without other symptoms. During transportation, her heart rate was 30 beats per minute, so 0.5 mg of atropine was given. On admission, the blood pressure was 92/51 mmHg, heart rate was 39 beats per minute, respiratory rate was 18/minute, tympanic temperature was 36.9ºC, and peripheral saturation was 95% (FiO_2_ 31%). After laboratory test results, hyperkalemia was noted (6.5 mEq/L); she also had mild hypernatremia (131 mEq/L) and acute kidney injury (creatinine 1.74 mg/dL; urea nitrogen 140 mg/dL). Her TSH was elevated (15.58 IU/mL) with normal free T4, and CRP was also elevated (44.9 mg/L). 

**Figure 1:**
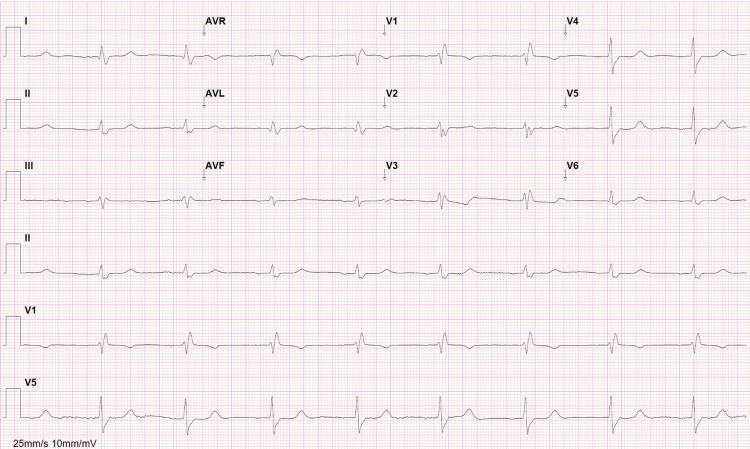
Electrocardiogram on hospital admission

EKG showed junctional rhythm as shown in Figure 1. Although QRS complexes were not narrow, QRS duration on a previous EKG was already 130 ms. The presentation and findings seemed consistent with BRASH syndrome; therefore, amlodipine was stopped, and we decided on a conservative strategy, treating hyperkalemia and also volume depletion with normal saline. The clinical and laboratory evolution was favorable, with supportive therapy only. All laboratory results on this admission and their evolution after five days can be found in Table 1.

**Table 1:** Laboratory findings on admission and day 5 hs-cTnT, High-sensitivity cardiac troponin T; CRP, C-reactive protein; TSH, thyroid-stimulating hormone.

Variables	Admission	Day 5	Normal range
Sodium (mmol/liter)	131	142	136–142
Potassium (mmol/liter)	6.5	4.6	3.5–5.0
Chloride (mmol/liter)	103	105	98–108
Bicarbonate (mmol/liter)	24		22–28
Urea nitrogen (mg/dl)	140	63	<50
Creatinine (mg/dl)	1.74	1.03	0.71–1.2
Glucose (mg/dl)	268	127	65–110
pH	7.26		7.35–7.45
pCO_2_ (mmHg)	54		33–48
pO_2_ (mmHg) - FiO_2_ 31%	94		>65
TSH (µIU/mL)	15.58		0.35–4.94
Free T4 (ng/dL)	0.95		0.70–1.48
hs-cTnT (ng/L)	15.3		<1.3
CRP (mg/L)	44.9		<0.5

## Discussion

The BRASH syndrome, which involves the presence of bradycardia, renal failure, AV nodal blockade, shock/hemodynamic instability, and hyperkalemia simultaneously, is the result of synergistic bradycardia due to the combination of hyperkalemia and medications that block the AV node. The most common trigger is hypovolemia [2].

Although hypothermia may have negatively influenced the blood gas results on the second presentation and also bradycardia, playing a significant role, the presentation cannot be fully explained by hypothermia alone and could itself be an adverse effect of the therapy with beta-blockers, especially after cold exposure. Similarly, although severe hypothyroidism should also be on the differential for the third presentation and must also have a significant role, the favorable evolution without specific treatment (no levothyroxine or corticotherapy was given) is against hypothyroidism alone.

The authors believe that this is a case of BRASH syndrome in a patient taking amlodipine triggered by hypovolemia due to aqueous diarrhea and consequent pre-renal renal failure and hyperkalemia, together with a drug that has an effect on the AV node (amlodipine) [6,7], and with hypothyroidism playing a role. We also believe that the patient's first hospitalization was due to BRASH syndrome related to beta-blocker use. Support treatment of each of the components separately (hyperkalemia and bradycardia, without invasive procedures like dialysis or pacemaker insertion) led to the resolution of the clinical problem. However, its recognition as a particular clinical entity could have led to a different choice of the anti-hypertensive treatment, other than one acting on the AV node, upon discharge from the previous hospitalization. According to the literature reviewed, this is the second report of BRASH syndrome that is related solely to amlodipine use [2,8].

## Conclusions

The case we report highlights the importance of recognizing BRASH syndrome as a particular entity in order to prevent its recurrence. We believe this to be an underdiagnosed syndrome that could eventually be better managed if clinicians were more aware of its existence, precipitants, and treatment.
